# Heterospecific interaction in two beetle species: Males with weapons decrease the reproductive success of species with weaponless males

**DOI:** 10.1002/ece3.11518

**Published:** 2024-06-18

**Authors:** Rui Onishi, Kentarou Matsumura

**Affiliations:** ^1^ Graduate School of Environmental, Natural Science, and Technology Okayama University Okayama Japan

**Keywords:** *Gnatocerus cornutus*, heterospecific interaction, male–male competition, sexual selection, *Tribolium castaneum*

## Abstract

Many species often show male–male combat for mating opportunities and resources within the species. Sexual selection through this radical combat leads to the evolution of males with exaggerated traits used as weapons, such as horns or mandibles, that often result in victory during combat. However, heterospecific interaction due to errors in species identification has often been observed, which results in decreased mating opportunities within the same species and fewer fertilized eggs. Males with exaggerated weapons may show dominance in resource acquisition over males without weapons and may decrease the reproductive success of the latter due to competition between the two. However, few studies have examined heterospecific interaction focusing on males with or without weapons. In this study, we investigated the effects of the male weapon on reproductive traits in heterospecific interaction in two species: the broad‐horned flour beetle (*Gnatocerus cornutus*), in which males have exaggerated weapon traits; and the red flour beetle (*Tribolium castaneum*), in which males have no weapon traits. Both species are closely related and use the same food resources. *G. cornutus* males interfered with the resource acquisition and reproductive opportunities of *T. castaneum* by attacking *T. castaneum*. The reproductive success of *T. castaneum* decreased when they cohabited with *G. cornutus* males. These findings show that male weapon traits, which are important for sexual selection within the same species, can also greatly influence reproduction in other species.

## Graphical abstract

Weapon traits possessed by males of many species play an important role in male–male combat for reproduction, but it was unclear whether they affect reproduction in other species. We compared the reproductive success of each other using *Gnatocerus cornutus*, whose males have the weapon, and its close relative, *Tribolium castaneum*, whose males do not have weapon. We found that the presence of *G. cornutus* males reduced the reproductive success of *T. castaneum*, but not vice versa.

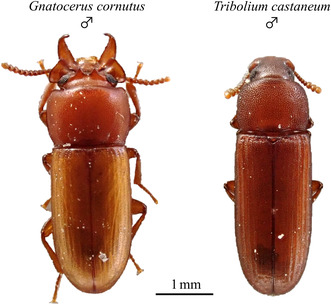

## 1 INTRODUCTION

Many species exhibit exaggerated sexual dimorphism used for courtship displays, such as the colorful tail feathers of the Indian peafowl, *Pavo cristatus* (Darwin, 1871); the large mandibles of Forrester's stag beetle, *Hexarthrius forsteri* (Otte & Stayman, 1979); and the interocular distance of the fly *Cyrtodiopsis whitei* (Burkhardt & de la Motte, 1988). Such sexual dimorphism has evolved through sexual selection (Darwin, 1871), in which selection pressure strongly affects a particular sex (often males) in order to increase reproductive success. In addition to intersexual selection, intrasexual selection, such as male–male competition for limited resources (e.g., mating opportunities with females or attracting females with food), is important for reproductive success. Exaggerated traits in males, such as horns or mandibles, have evolved through intrasexual selection as weapons for male–male combat (Andersson, 1994; Eberhard, 1979, 1980; Palmer, 1978). Thus, both intersexual selection within a species and intrasexual selection are important for the evolution of reproductive traits.

Studies have shown that if reproduction‐related morphological and behavioral traits are similar, mating occurs even between different species because of incomplete reproductive isolation (Burdfield‐Steel & Shuker, 2011). However, interspecies mating decreases mating opportunities within the same species and lowers fitness due to genetic incompatibility and the production of sterile offspring. Direct interference between species in the reproductive process can occur at various stages of interspecies courtship and produce infertile hybrids (Gröning & Hochkirch, 2008). This phenomenon is observed across a wide range of taxa. For example, in plants, studies have reported heterospecific reproduction between the native species *Taraxacum japonicum* and the invasive species *Taraxacum officinale* in Japan (Takakura et al., 2009). Even if sufficient pollen from the same species is present, the fertilization rate of *Taraxacum japonicum* decreases when *Taraxacum officinale* pollen attaches to the stigma (Matsumoto et al., 2010). In contrast, *Taraxacum officinale* reproduces asexually and is not affected by *Taraxacum japonicum* pollen. Guppy ( *Poecilia reticulata* ) males may perform forced copulation with female mosquitofish (*Gambusia affinis*), which decreases the number of mosquitofish offspring (Fujimoto et al., 2021; Tsurui‐Sato et al., 2019). In interspecies mating between the Asian tiger mosquito (*Aedes albopictus*) and the yellow fever mosquito (*A. aegypti*), male *A. albopictus* mate and transfer sperm to female *A. aegypti* (Nasci et al., 1989). This heterospecific interaction is believed to be caused by differences in the ability of the *A. albopictus* males to distinguish between conspecific and heterospecific females because female receptivity is not different between the two species (Manning, 1959). Heterospecific interaction leads to a decrease in fitness across generations, so it is important to examine this interaction for evolutionary biology.

Studies on interspecies interaction have focused on intersexual selection, such as male courtship toward females of different species and female acceptance of mates. However, coexistence of species and the ability of males to mate with females of a different species lead to competition with males of other species for resources and mating opportunities, potentially affecting mating opportunities within their own species. If different species are phenotypically similar, the males of one species may misidentify dissimilar males as conspecific males and engage in male–male combat or even misidentify dissimilar females as conspecific males and attack them (Gröning & Hochkirch, 2008). In addition, males with exaggerated intrasexual selection traits associated with male–male combat, such as weapons, have a greater advantage in interspecies interaction, and these traits may influence heterospecific interaction (Shuker & Burdfield‐Steel, 2017). However, few studies have investigated the effects of intrasexual selection traits on heterospecific interaction (Gröning & Hochkirch, 2008).

**FIGURE 1:**
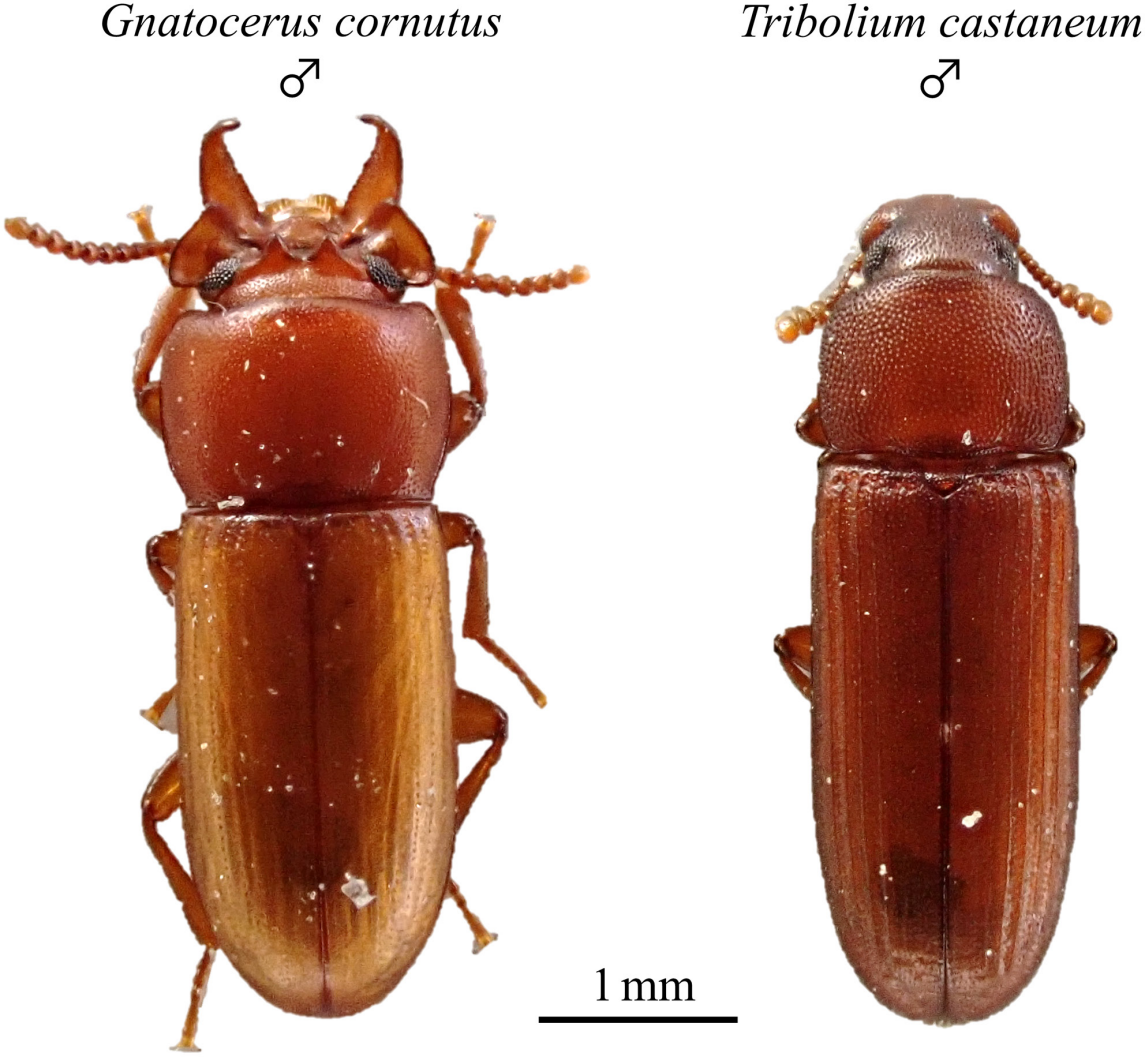
Males of *Gnatocerus cornutus* and *Tribolium castaneum*. *Gnatocerus cornutus* male has exaggerated mandible, whereas *T. castaneum* male does not have exaggerated mandible.

In this study, we investigated the effects of intrasexual selection traits on heterospecific interaction, focusing on reproductive traits in the broad‐horned flour beetle (*Gnatocerus cornutus*), in which the male has exaggerated weapon traits, and the red flour beetle (*Tribolium castaneum*), in which the male does not have exaggerated weapon traits (Figure 1). These two closely related species have similar habitats and life histories (Hinton, 1942; Pray & Goodnight, 1995; Zakladnoi & Ratanova, 1987), so interactions between them may actually occur in the field. *G. cornutus* males use their large mandibles as weapons during male–male combat, and males with larger weapons have a greater advantage when competing for resources and mating opportunities. The exaggerated weapon traits of *G. cornutus* males have evolved through intrasexual selection (Okada et al., 2006). The intensity of male–male combat may be weaker in *T. castaneum* than in *G. cornutus*. *G. cornutus* uses a resource defense strategy (Okada et al., 2006), so males fight each other even in the absence of females. *G. cornutus* males may attack *T. castaneum* and may interfere with their reproduction. If *G. cornutus* and *T. castaneum* cohabitate and their males compete for resources and mating opportunities, heterospecific interaction may occur. As *G. cornutus* males are more advantaged during male–male combat, *G. cornutus* males may decrease the reproductive success of *T. castaneum*. In both beetle species, females accept mates relatively actively without showing any significant refractory period (Fedina & Lewis, 2008; Okada et al., 2014). However, although several studies have investigated heterospecific interaction in *T. castaneum* (Kishi, 2014), there is no study on heterospecific interaction in *G. cornutus*.

In this study, we conducted three experiments on *G. cornutus* and *T. castaneum*. In experiment 1, we paired *G. cornutus* and *T. castaneum* males and observed them for any aggressive behavior toward each other. We also observed the mating behavior in the two species to evaluate whether the presence of a heterospecific male affects the mating behavior of *G. cornutus* or *T. castaneum* pairs. In experiment 2, we investigated the effect of the presence of heterospecific males on reproductive traits in conspecific pairs before mating by determining the number of eggs laid and the hatching rates when conspecific pairs were cohabitated with heterospecific males. In experiment 3, we investigated the effect of the presence of heterospecific males on reproductive traits after mating by determining the number of eggs laid and the hatching rates when a heterospecific male cohabited with a female that had mated with a conspecific male. We hypothesized that (1) if *G. cornutus* males exhibit aggressive behavior toward *T. castaneum* males, the reproductive success of *T. castaneum* will decrease because the mating of *T. castaneum* is disturbed in experiment 2; (2) if *G. cornutus* shows aggressive behavior toward *T. castaneum* females, the oviposition of *T. castaneum* females may be disturbed in experiment 3; and (3) conversely, if *G. cornutus* males display mating behavior toward *T. castaneum* females, the hatching rate of *T. castaneum* will decrease.

## 2 MATERIALS AND METHODS

### 2.1 Insect culture

The stock cultures of *G. cornutus* and *T. castaneum* used in this study have been maintained in the laboratory for >40 years (Miyatake et al., 2004; Okada et al., 2006). Both beetle species were cultured on wheat flour with 5% beer yeast. The cultures were incubated at 28°C in a 16 h/8 h light/dark cycle. For all experiments, individuals from both species were isolated before they became adults because the *G. cornutus* male's behavior changes when it loses in male–male combat (Okada & Miyatake, 2010) and because a high larval density suppresses pupation (Tsuda & Yoshida, 1985). Therefore, each final instar larva was placed individually in a well plate. In experiments 2 and 3, abundant wheat flour was provided to avoid competition for food resources.

### 2.2 Measurement of reproductive traits

#### 2.2.1 Observation of behaviors of heterospecific males

In experiment 1, we observed the behavior of a pair of *G. cornutus* and *T. castaneum* males placed in the same space. Briefly, a virgin *G. cornutus* male and a virgin *T. castaneum* male with no fighting experience were placed simultaneously in a cylindrical plastic container (16 mm in diameter, 10 mm in depth) (i.e., *G. cornutus*♂ vs. *T. castaneum* ♂). We observed the pair for 15 min, during which time we recorded the number of attacks on the rival male. An attack was defined as an action taken by the one male species in an attempt to lift its rival off the substrate by placing its mandibles under the rival's body (Okada et al., 2006). Although *G. cornutus* males often attacked *T. castaneum*, because *T. castaneum* does not possess weapons and does not have attacking behavior within their repertoire, we cannot observe aggressive behavior of *T. castaneum* for *G. cornutus* males. Therefore, the number of times *T. castaneum* escaped from *G. cornutus* after the attack by *G. cornutus* male was recorded. In total, we observed 10 pairs of *G. cornutus* and *T. castaneum* males.

We also investigated whether the presence of a heterospecific male affects the mating frequency and duration in *T. castaneum* and *G. cornutus*. One conspecific male and female pair was placed in a cylindrical plastic container (16 mm in diameter, 10 mm in depth) as the control group (i.e., *G. cornutus* ♂♀ or *T. castaneum* ♂♀), whereas in the treatment group, an additional heterospecific male was added to the conspecific pair (i.e., *G. cornutus*♂ + *T. castaneum*♂♀ or *T. castaneum*♂ + *G. cornutus*♂♀). We observed the groups for 15 min, during which time we recorded the mating frequency and duration (the time from when the male mounts the female and begins rubbing its legs against the side of the female's body until the male leaves the female). The mating style of both species is similar. Mating initiation was defined as the male mounting the female and quickly rubbing its legs against the side of the female's body (Okada et al., 2014; Wojcik, 1969). In total, we observed 40 pairs (10 pairs each for *G. cornutus* ♂♀, *T. castaneum* ♂♀, *G. cornutus* ♂ + *T. castaneum* ♂♀, and *T. castaneum* ♂ + *G. cornutus* ♂♀).

#### 2.2.2 Effect of coexistence with heterospecific males before mating: number of eggs laid and hatching rate

In experiment 2, we aimed to investigate whether the presence of a heterospecific male affects the number of eggs laid and the hatching rate in *G. cornutus* and *T. castaneum* (before mating). We put a pair of virgin male and virgin female of the same species in a cylindrical plastic container (30 mm in diameter, 10 mm in depth) with food (i.e., *G. cornutus* ♂♀ or *T. castaneum* ♂♀). To investigate the effects of heterospecific males, one heterospecific virgin male was placed in container containing a pair of same species (i.e., *G. cornutus* ♂ + *T. castaneum* ♂♀ or *T. castaneum* ♂ + *G. cornutus* ♂♀). To investigate the effect of increasing the number of males, we also created a group in which one male of the same species was added (i.e., *G. cornutus* ♂♂♀ or *T. castaneum* ♂♂♀). We collected eggs every 4 days and confirmed hatching every 2 days. Egg collection was conducted five times. In total, we determined the number of eggs laid and the hatching rate for 100 pairs (10 pairs each for *G. cornutus* ♂♀ and *T. castaneum* ♂♀; 20 pairs each for *G. cornutus*♂ + *T. castaneum* ♂♀, *T. castaneum* ♂ + *G. cornutus* ♂♀, *G. cornutus* ♂♂♀, and *T. castaneum* ♂♂♀).

#### 2.2.3 Effect of coexistence with heterospecific males after mating: number of eggs laid and hatching rate

In experiment 3, we aimed to investigate whether the presence of a heterospecific male affects the number of eggs laid and the hatching rate in *T. castaneum* and *G. cornutus* (after mating). First, a pair of conspecific male and female was allowed to cohabitate for 24 h in a cylindrical plastic container (16 mm in diameter, 10 mm in depth). Next, the pair was transferred to a cylindrical plastic container (30 mm in diameter, 10 mm in depth) with food, and the female was allowed to lay eggs (i.e., *G. cornutus* ♂♀ or *T. castaneum* ♂♀). To investigate the effects of heterospecific males, one heterospecific virgin male was placed in container containing a female that previously mated (i.e., *G. cornutus* ♂ + *T. castaneum* ♀ or *T. castaneum* ♂ + *G. cornutus* ♀). To investigate the effect of replaced male, we also created a group in which one male of the same species was replaced (i.e., *G. cornutus* ♂ + *G. cornutus* ♀ or *T. castaneum* ♂ + *T. castaneum* ♀). We collected eggs every 4 days and confirmed hatching every 2 days. Egg collection was conducted five times. In total, we determined the number of eggs laid and the hatching rate for 100 pairs (10 pairs each for *G. cornutus* ♂♀ and *T. castaneum* ♂♀; 20 pairs each for *G. cornutus* ♂ + *T. castaneum* ♀, *T. castaneum* ♂ + *G. cornutus* ♀, *G. cornutus* ♂ + *G. cornutus* ♀, and *T. castaneum* ♂ + *T. castaneum* ♀).

### 2.3 Statistical analysis

We analyzed the number and duration of copulation data using a generalized linear mixed model (GLMM) with treatment as a fixed effect and the experiment day as a random effect (distribution, link function, and Akaike's information criterion of each model were described in Table S1). To analyze the results for the number of eggs, we used GLMM and Bonferroni correction to determine whether any significant difference was observed with the GLMM method (distribution, link function, and Akaike's information criterion of each model were described in Table S1). To analyze the results for the hatching rate, we used a GLMM with binomial distribution, but it was overdispersed. Therefore, we used the estimated dispersion parameter to correct the variance (Collett, 2002; Faraway, 2016). All statistical analyses were performed using R ver. 3.4.3 (R Core Team, 2017) and the *car* package ver. 3.0.11 in R (Fox & Weisberg, 2018).

## 3 RESULTS

**FIGURE 2:**
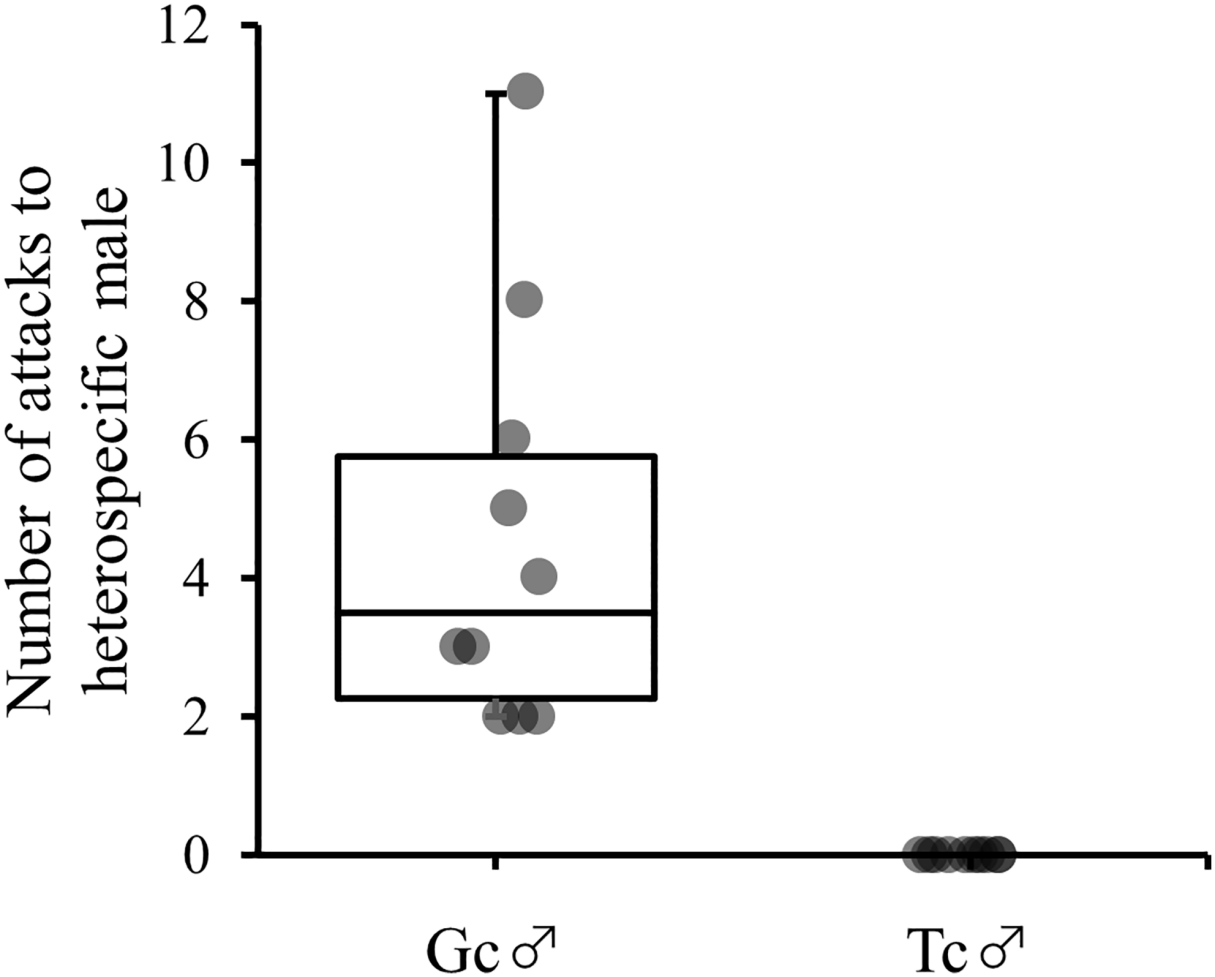
Box plots of number of attacks on heterospecific male by Gc (*Gnatocerus cornutus*) male and Tc (*Tribolium castaneum*) males, respectively.

**FIGURE 3:**
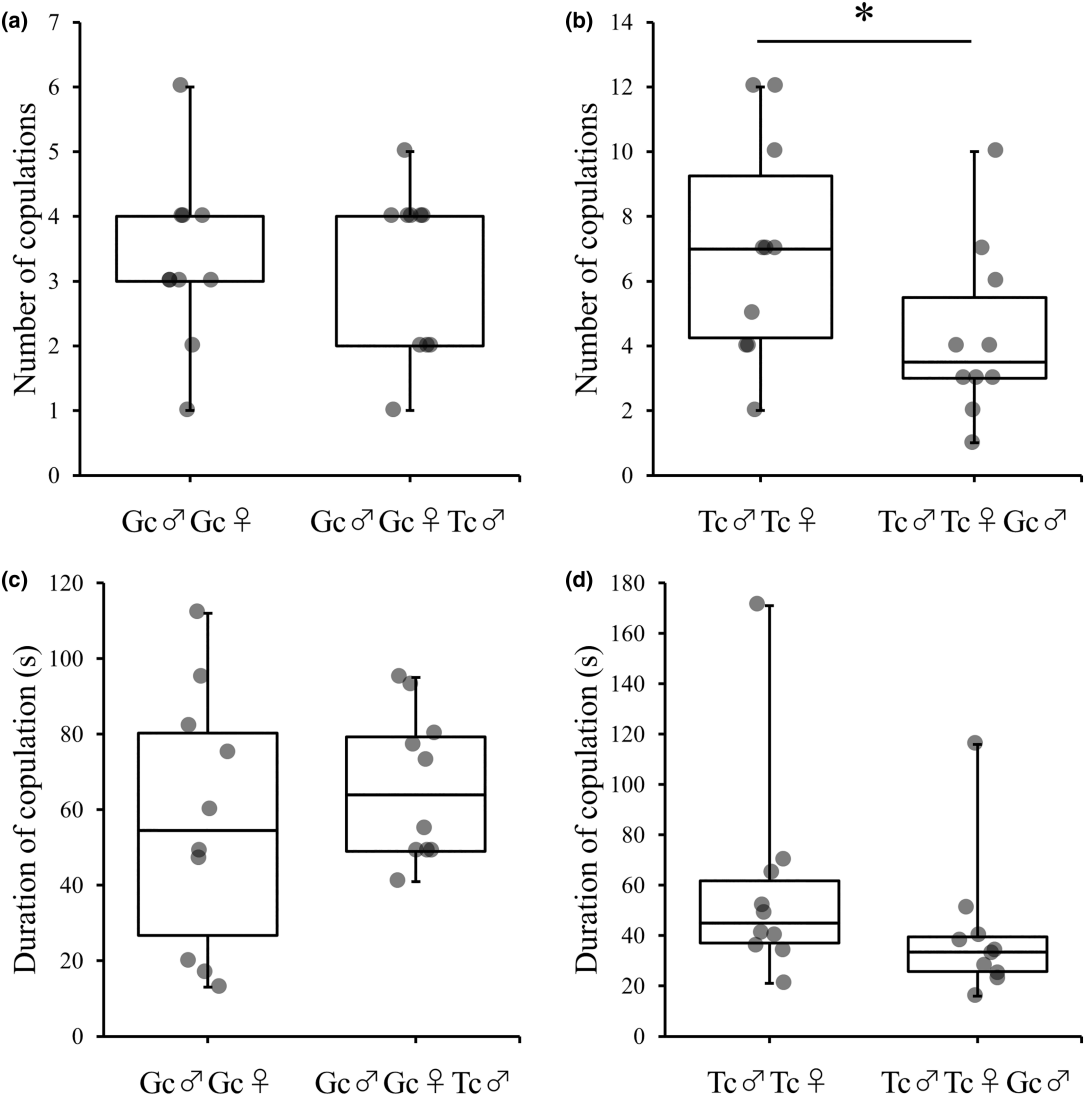
Effects of exist of heterospecific male on number of copulations (a and b) and duration of copulation (c and d). Gc and Tc show *Gnatocerus cornutus* and *Tribolium castaneum*, respectively. (a and c) show effects of existence of *T. castaneum* male for *G. cornutus* pair, and (b and d) show effects of existence of *G. cornutus* male for *T. castaneum* pair, respectively.

All *G. cornutus* males exhibited aggressive behavior toward *T. castaneum* males, whereas all *T. castaneum* males did not exhibit aggressive behavior toward *G. cornutus* males (Figure 2). Figure 3 shows the mating frequency and the average mating duration in the presence of heterospecific males. In *G. cornutus* pairs, there was no significant effect of the presence of *T. castaneum* males in both the number of copulations ($\chi_{1,17}^{2}$ = 0.03, *p* = .864; Figure 3a) and the duration of copulation ($\chi_{1,17}^{2}$ = 0.53, *p* = .466; Figure 3c). Conversely, the mating frequency of *T. castaneum* pairs significantly decreased in the presence of *G. cornutus* males compared to the mating frequency in the absence of *G. cornutus* males ($\chi_{1,17}^{2}$ = 7.29, *p* = .007; Figure 3b); however, there was no significant difference in the mating duration ($\chi_{1,17}^{2}$ = 1.18, *p* = .277; Figure 3d).

**FIGURE 4:**
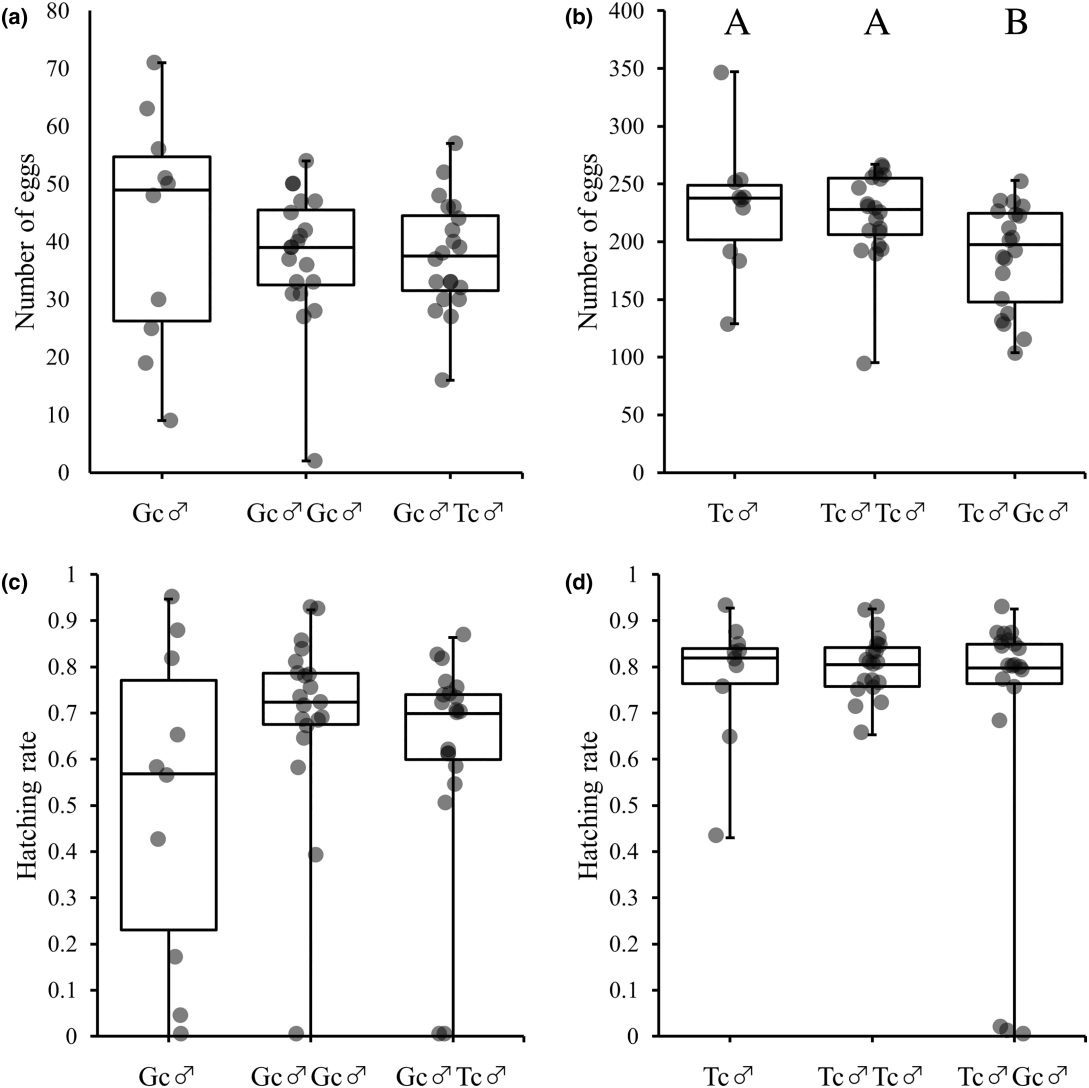
Results of experiment 2 that investigated effects of the presence of heterospecific males prior to copulation on the number of eggs (a and b) and hatching rate (c and d). Gc and Tc show *Gnatocerus cornutus* and *Tribolium castaneum*, respectively. Gc♂ and Tc♂ indicate populations cohabited with conspecific pairs of *G. cornutus* or *T. castaneum*. Gc♂Gc♂ and Gc♂Tc♂ indicate populations in which two *G. cornutus* males or one *G. cornutus* male and one *T. castaneum* male cohabited with a *G. cornutus* female. Tc♂Tc♂ and Tc♂Gc♂ indicate populations in which two *T. castaneum* males or one *T. castaneum* male and one *G. cornutus* male cohabitated with a *T. castaneum* female (detailed in Section 2). (a and c) show effects of existence of *T. castaneum* male for *G. cornutus* pair, and (b and d) show effects of existence of *G. cornutus* male for *T. castaneum* pair, respectively.

In results of experiment 2, in *G. cornutus* pairs, there was no significant effect of the presence of *T. castaneum* males in both the number of eggs ($\chi_{1,237}^{2}$ = 2.06, *p* = .357; Figure 4a) and the hatching rate (*F*_2,47_ = 1.42, *p* = .251; Figure 4c). Conversely, the number of eggs laid by *T. castaneum* females significantly decreased in the presence of *G. cornutus* males compared to the number of eggs laid in the absence of *G. cornutus* males ($\chi_{1,242}^{2}$ = 10.38, *p* = .006; Figure 4b); however, there was no significant difference in the hatching rate (*F*_2,47_ = 0.86, *p* = .431; Figure 4d).

**FIGURE 5:**
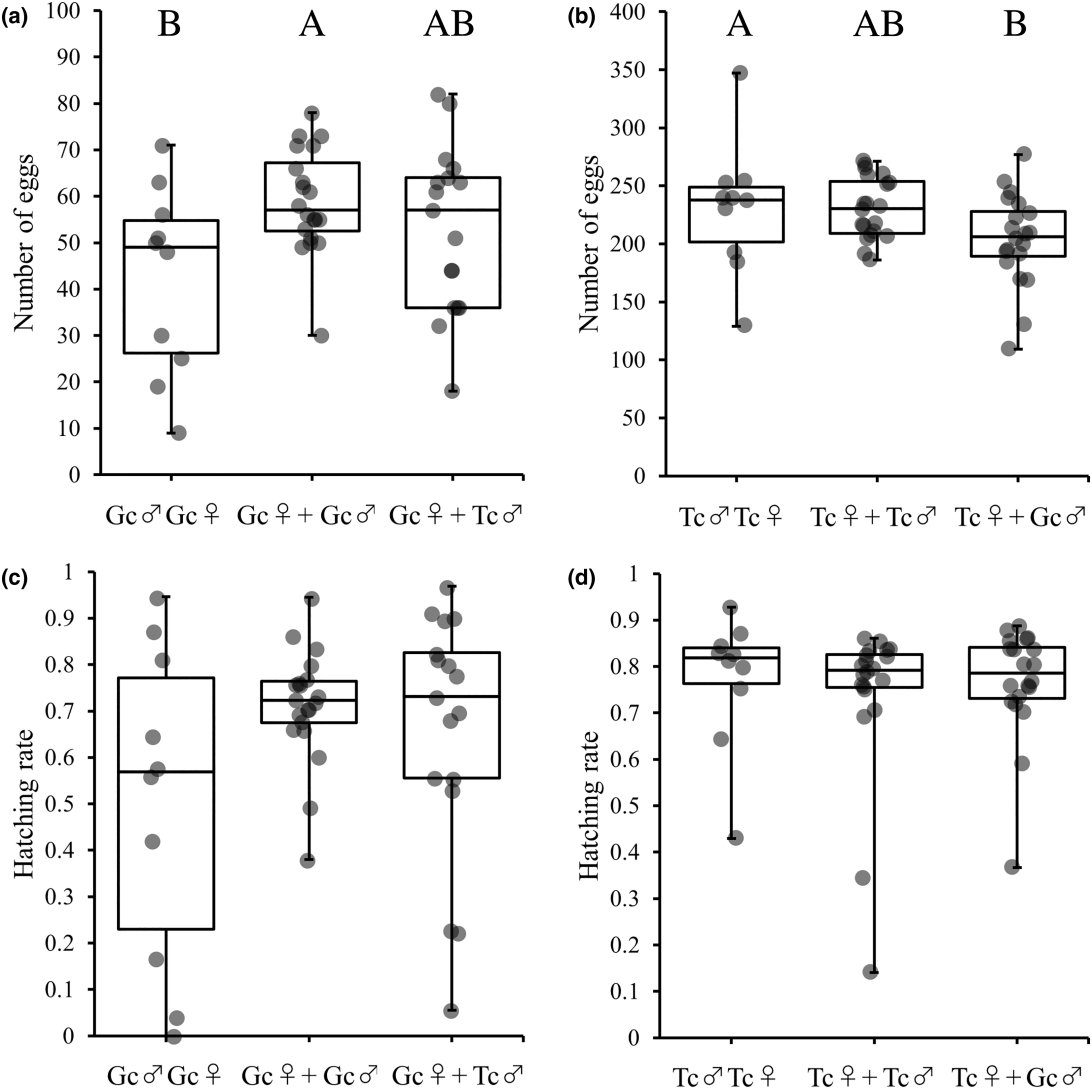
Results of experiment 3 that investigated effects of the presence of heterospecific males after copulation on the number of eggs (a and b) and hatching rate (c and d). Gc and Tc show *Gnatocerus cornutus* and *Tribolium castaneum*, respectively. Gc♂Gc♀ and Tc♂Tc♀ indicate populations that cohabited with conspecific pairs of *G. cornutus* or *T. castaneum*, respectively. Gc♀ + Gc♂ and Tc♀ + Tc♂ indicate populations that males are replaced with conspecific males and cohabited after mating in *G. cornutus* pair or *T. castaneum* pairs, respectively. Gc♀ + Tc♂ and Tc♀ + Gc♂ indicate populations that males are replaced with heterospecific males and cohabited after copulation in *G. cornutus* or *T. castaneum* pairs, respectively (detailed in Section 2). (a and c) show effects of existence of *T. castaneum* male for *G. cornutus* female that copulated previously, and (b and d) show effects of existence of *G. cornutus* male for *T. castaneum* female that copulated previously, respectively.

In results of experiment 3, there was no significant difference in the number of eggs *G. cornutus* female ($\chi_{1,224}^{2}$ = 3.01, *p* = .222; Figure 5a). Furthermore, there was no significant difference in the hatching rate (*F*_2,44_ = 0.60, *p* = .553; Figure 5c). In addition, the number of eggs laid by *T. castaneum* females significantly decreased in the presence of *G. cornutus* males compared to the number of eggs laid in the absence of *G. cornutus* males ($\chi_{1,242}^{2}$ = 8.05, *p* = .018; Figure 5b); however, there was no significant difference in the hatching rate (*F*_2,47_ = 0.29, *p* = .7511; Figure 5d).

## 4 DISCUSSION

Our results revealed heterospecific interaction in the reproduction between *T. castaneum* and *G. cornutus*, where the reproductive success of *T. castaneum* was unilaterally negatively affected by *G. cornutus* males. *G. cornutus*, in which males have exaggerated weapons, negatively affected the reproduction of *T. castaneum*, in which males do not have weapons. Although several studies have investigated heterospecific interaction focusing on the reproductive traits between males and females, our study is the first to investigate heterospecific interaction focusing on intrasexual selected traits (i.e., weapons used in male–male competition). Therefore, these results are significant from an evolutionary ecology point of view.

The results of experiment 1 showed that *G. cornutus* males display unilateral aggressive behavior toward *T. castaneum* males, indicating that *G. cornutus* males are more dominant than *T. castaneum* males in securing food and space for mating. The mating frequency of *T. castaneum* male and female pairs in the presence of *G. cornutus* males significantly decreased compared to that in the absence of *G. cornutus* males. This shows that the attack on *T. castaneum* by *G. cornutus* males is one of the factors that decreases the mating frequency of *T. castaneum*. *G. cornutus* males were also observed to attack *T. castaneum* females (Onishi & Matsumura, unpublished data), indicating that *G. cornutus* males are more likely to attack both sexes of heterospecific beetles. The presence of *T. castaneum* males did not affect the mating frequency of *G. cornutus* male and female pairs, suggesting that the negative effect on mating frequency in *T. castaneum* is unilateral. The mating duration was not affected by the presence of *G. cornutus* males, indicating that the fighting behavior of *G. cornutus* males does not affect *T. castaneum* pairs during mating but rather decreases the opportunity for *T. castaneum* mating. However, *G. cornutus* males were not observed to display courtship behavior toward *T. castaneum* females, suggesting that heterospecific mating does not occur.

The results of experiments 2 and 3 showed that the presence of *G. cornutus* males significantly decreased the number of eggs laid by *T. castaneum* females, both before and after mating, whereas the presence of *T. castaneum* males had no significant effect on the number of eggs laid by *G. cornutus* females. Moreover, the presence of heterospecific males did not affect the hatching rate in either species. These results may have been influenced by cannibalism, which often occurs in both species. However, cannibalism is often primarily directed at pupae in both species (Ozawa et al., 2015). In addition, the eggs were collected every 4 days, and the larvae were removed every 2 days before they could develop, making it unlikely that the decrease in egg laying was due to cannibalism by the first instar larvae. Thus, the effects of cannibalism on our results may be small. The decrease in the number of eggs laid can be attributed to the indiscriminate aggressive behavior of *G. cornutus* males observed in experiment 1. For example, *G. cornutus* males may directly inhibit the oviposition of *T. castaneum* females, or the stress caused by attacks from *G. cornutus* males may decrease the number of eggs laid. Future studies should investigate these observations in detail.

Previous studies have reported that the *T. castaneum* female used the sperm of the last‐mated male for fertilization (Lewis et al., 2005) and that the hatching rate decreased when interspecific mating occurs with the closely related species *T. confusum* (Birch et al., 1951). Our results showed no significant difference in the hatching rate between control and treatment groups. In addition, both experiments 2 (*G. cornutus* females were cohabitated with *T. castaneum* and *G. cornutus* males) and 3 (only *T. castaneum* males were cohabitated with *G. cornutus* females) had no effect on the hatching rate. This finding suggests that heterospecific mating does not occur between *G. cornutus* and *T. castaneum*. Conversely, in *Callosobruchus* species, heterospecific mating does not lead to a decrease in the hatching rate, and only male courtship of heterospecific females decreases the frequency of oviposition (Kyogoku, 2021; Kyogoku & Nishida, 2013). Therefore, it is not possible to assert based on only our results that heterospecific mating does not occur between *G. cornutus* and *T. castaneum*. More careful observation of heterospecific mating between the two beetle species is required.

The negative effects of heterospecific interaction on one species will eventually competitively eliminate that species. This phenomenon, called reproductive interference, has been studied extensively (Burdfield‐Steel & Shuker, 2011). Our results also suggest that *T. castaneum* will eventually be competitively eliminated by *G. cornutus* via reproductive interference. However, because this study did not examine fitness among generations, it is difficult to discuss reproductive interference between *G. cornutus* and *T. castaneum*. Moreover, although the number of eggs laid by *T. castaneum* decreased, the number of eggs laid by *T. castaneum* was still higher compared to *G. cornutus* (Figures 4a,b and 5a,b). However, this is not a basis for predicting that *T. castaneum* will be competitively eliminated by *G. cornutus*, and it is necessary to investigate the reproductive interference of the two beetles species in more detail in the future. In *T. castaneum*, there have been many studies on heterospecific interaction between two species with another relative species, *T. confusum* (Begon & Townsend, 2021; Cain et al., 1969). Because *T. castaneum* larvae prey on *T. confusum* eggs and larvae, it has been suggested that *T. castaneum* is more dominant in intraguild predation (Dawson & Lerner, 1966; Goodnight & Craig, 1996; Inouye & Lerner, 1965; Park et al., 1964). On the other hand, although *T. castaneum* males prefer to mate with conspecific females, *T. confusum* males also actively attempt to mate with *T. castaneum* females. Therefore, *T. castaneum* is inferior in reproductive interference (Birch et al., 1951; Lloyd & Park, 1962; Serrano et al., 2000). Thus, between these two species, intraguild cannibalism and reproductive interference are both asymmetric and counterbalanced (Kishi, 2014). In this study, we observed an interaction between the adult stages of *G. cornutus* and *T. castaneum*, but it is also important to investigate intraguild cannibalism during the larval stage. Furthermore, In the male–male combat by *G. cornutus*, the first stage of the fight was often to put each other's mandible into each other and to show behavior to ascertain the opponent (RO personal observation). Then, after a while, the struggle escalated as he grabbed the opponent with his mandibles and lifted him up. Thus, when *G. cornutus* encounter *T. castaneum* lacking mandible, the *G. cornutus* may not recognize the *T. castaneum* as a male of the same species. Therefore, the *G. cornutus* may have attacked *T. castaneum* not as a reproductive rival but as a resource defense rival.

Many species engage in male–male combat, also known as intrasexual selection (Andersson, 1994). In *G. cornutus*, males use their exaggerated mandibles in male–male combat to fight over resources and mating opportunities with females (Okada et al., 2006). In this study, we found that male weapon traits used in male–male combat in intraspecific competition also negatively affect the reproductive traits in other species. Therefore, focusing on such exaggerated sexual traits is also important in considering heterospecific interaction.

## AUTHOR CONTRIBUTIONS

**Rui Onishi:** Investigation (lead); writing – original draft (lead). **Kentarou Matsumura:** Conceptualization (equal); data curation (equal); formal analysis (equal); funding acquisition (equal); investigation (supporting); methodology (equal); project administration (equal); resources (equal); software (equal); supervision (equal); validation (equal); visualization (equal); writing – original draft (supporting); writing – review and editing (equal).

## CONFLICT OF INTEREST STATEMENT

The authors declare no conflict of interest.

## Supporting information


Data S1:

Table S1:

## Data Availability

Data are available in supplementary material.
