# Supplementary material for: Heterospecific interaction in two beetle species: Males with weapons decrease the reproductive success of species with weaponless males
Source: Ecol Evol. 2024 Jun 18;14(6):e11518. doi: 10.1002/ece3.11518 (PMC11184211; doi:10.1002/ece3.11518)
Supplement: Supplementary file 2 — Table S1: [file ECE3-14-e11518-s002.docx]

**Table S1**. Distribution, link function, Akaike’s information criterion (AIC) of each model.

| Experiment | Trait | Beetle | Distribution | Link | AIC |
| --- | --- | --- | --- | --- | --- |
| 1 | Number of matings | Tc | Poisson | sqrt | 99.1 |
|  |  | Gc | Gaussian | identity | 73.7 |
|  | Duration of mating | Tc | Gaussian | identity | 192.7 |
|  |  | Gc | Gaussian | identity | 183.6 |
| 2 | Number of matings | Tc | Gaussian | identity | 1953.6 |
|  |  | Gc | Gaussian | identity | 1265.6 |
| 3 | Number of matings | Tc | Gaussian | identity | 1922.1 |
|  |  | Gc | Gamma | identity | 1229.9 |
